# Extraskeletal myxoid chondrosarcoma with massive pulmonary metastases

**DOI:** 10.1186/s13569-018-0108-8

**Published:** 2018-12-05

**Authors:** Luca Paoluzzi, Munir Ghesani

**Affiliations:** 1 Department of Medicine, Sarcoma Medical Oncology, NYU Langone Medical Center, 160 East 34th Street, New York, NY 10016 USA; 2 Department of Radiology, NYU Langone Medical Center, New York, NY USA

**Keywords:** Extraskeletal myxoid chondrosarcoma, Pazopanib, NR4A3, Sarcoma

## Abstract

**Background:**

Extraskeletal myxoid chondrosarcoma (EMC) is a rare malignant mesenchymal neoplasm of uncertain differentiation characterized by rearrangements of the NR4A3 gene. EMC often affects adults around the age of 50 and arise in the deep tissues of the proximal extremities and limb girdles. EMC is characterized by indolent growth rate but strong tendency to local recurrence and metastatic spread. No systemic treatment is specifically approved by the FDA for this disease and surgery has been traditionally the only potentially curative strategy.

**Case presentation:**

A 41-year-old Caucasian woman originally presented with a 14.8 cm left thigh mass. She was managed with wide local resection but after 2 years she developed recurrent disease in the pelvis and in the lungs; the lung involvement was characterized by innumerable nodules without any significant respiratory symptoms. After failing three clinical trials, she experienced prolonged disease control while on treatment with the tyrosine kinase inhibitor (TKI) pazopanib and radiation therapy delivered to the pelvic lesion. Dose reduction of pazopanib due to severe diarrhea was followed by rapid disease progression in the pelvis requiring vascular stenting; increase in tumor growth after discontinuation of a TKI has been described in other malignancies and is a possibility in this specific patient.

**Conclusion:**

While surgical management of EMC with or without radiation therapy is still the preferable approach when feasible, small series support the use of tyrosine kinase inhibitors and possible new immunotherapies in selected patients. Basket trials focusing on diseases with unique genomic features such as EMC will hopefully provide a better understanding of new options for care.

## Background

Extraskeletal myxoid chondrosarcoma (EMC) is a malignant mesenchymal neoplasm of uncertain differentiation characterized by rearrangements of the NR4A3 gene. It accounts for less than 3% of soft tissue sarcomas (STS); usually occurs in adults with a median age of 50 years and a male to female ratio of 2:1. Most EMCs arise in the deep tissues of the proximal extremities and limb girdles with thigh being the most common site. Less common sites include the trunk, head and neck, abdomen, pelvis, paraspinal soft tissue and foot; rare locations include cranium, retroperitoneum, pleura and bone [1].

EMC is an aggressive malignancy with local recurrence rates in the 37–48% range and metastases occurring in about 50% of the cases; prolonged survival despite metastatic disease is not uncommon however. Retrospective series report survival rates at 5 years of 82–90%, at 10 years of 65–70%, at 15 years of 58–60%. Adverse prognostic factors include old age, tumor size more than 10 cm, proximal location; additional factors may include increased cellularity and atypia or presence of rhabdoid cells [1]. Current treatment is based on surgical resection that can be curative in some patients.

## Case presentation

**Fig. 1:**
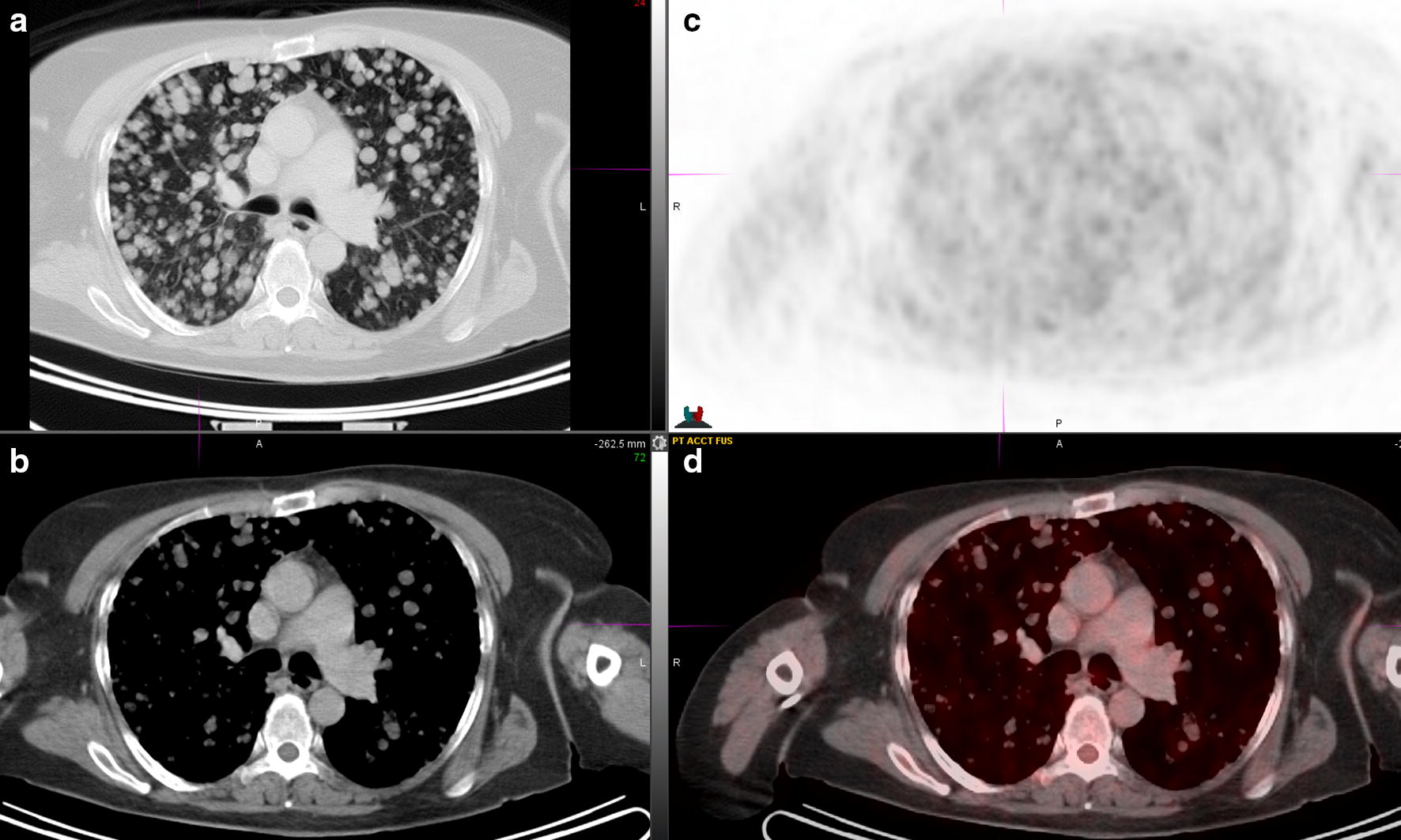
PET/CT at the time pazopanib was started. **a** CT lung window; **b** CT mediastinal window; **c** PET; **d** fusion CT and PET

**Fig. 2:**
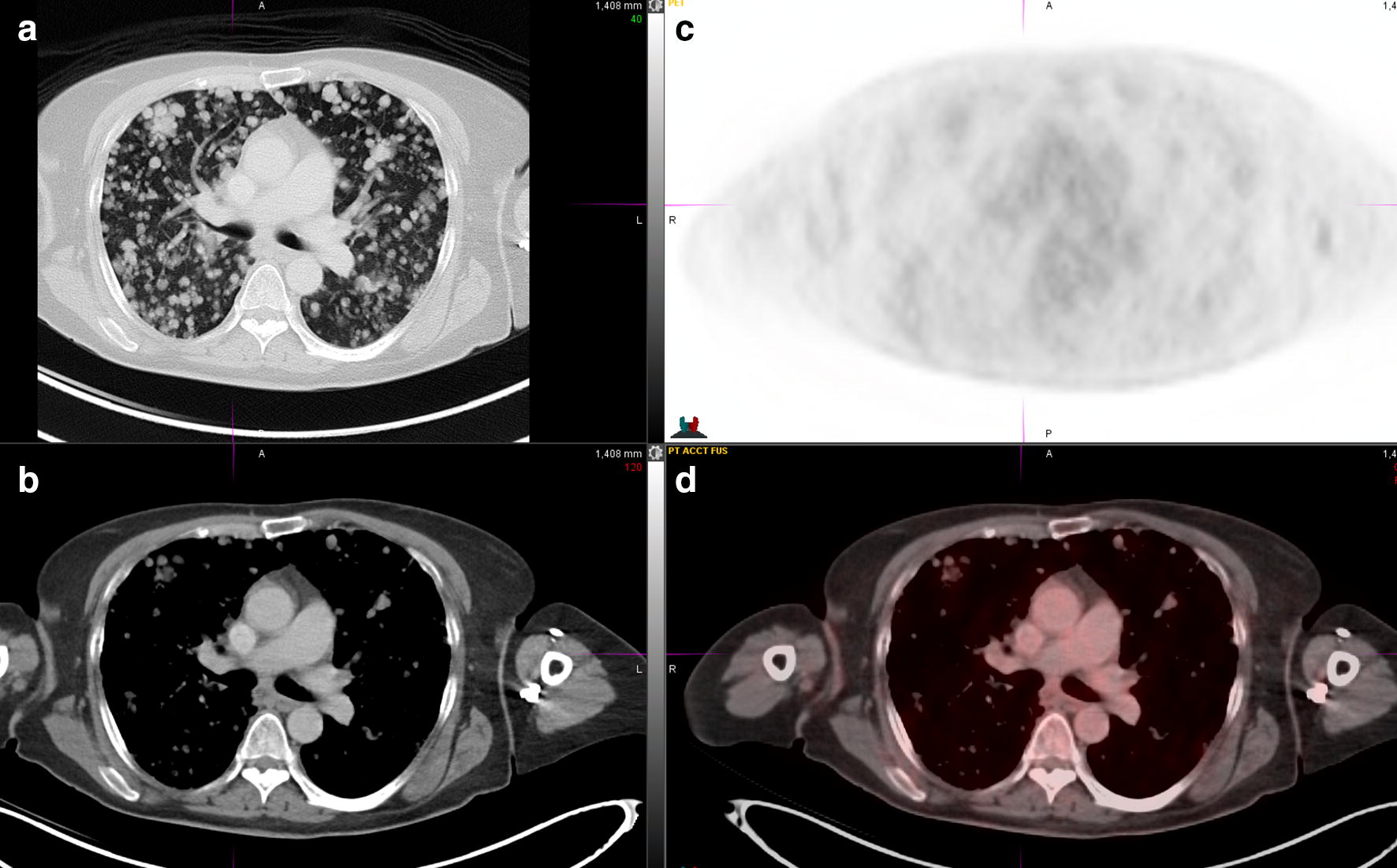
PET/CT 4 years since pazopanib was started. **a** CT lung window; **b** CT mediastinal window; **c** PET; **d** fusion CT and PET

A 41-year-old Caucasian woman presented with a mass in the distal part of her medial left thigh for which she underwent surgical resection; pathology was consistent with an extraskeletal myxoid chondrosarcoma, tumor size was 14.8 cm in greatest dimension, the closest margin was less than 0.5 mm from the tumor. Next generation sequencing (NGS) was performed using a commercially available platform; it showed the presence of the EWSR1–NR4A3 translocation, mutation in the tumor suppressor gene CHEK2, stable microsatellite status and low tumor mutational burden (TMB, 5 mutations/Mb). Mutations of unknown significance were found in genes related to known oncogenic pathways such as MAPK (MAP3K1 and 6), AKT (ZNF217) and NF-kB (IKBKE) or genes involved in chromatin remodeling (BCOR, MLL2), metabolism (AR), angiogenesis (GPR124) and immune response (PDCD1LG2). Patient was subsequently followed with surveillance imaging; 2 years later she was found to have a recurrence in the original surgical site; chest imaging showed multiple lung nodules. During the following 5 years the patient was enrolled sequentially into 3 clinical trials testing the following drugs: brivanib, a vascular endothelial growth factor receptor 2 (VEGF2) inhibitor, KW-2450, an insulin-like growth factor 1 receptor and insulin receptor (IGF-R1/IR) tyrosine kinase inhibitor (TKI) and PTC299, a vascular endothelial growth factor (VEGF) inhibitor. After further progression of disease in the left thigh, left pelvis, lung and liver, she was started on the TKI pazopanib. At that time the chest imaging was remarkable for massive infiltration by innumerable lung lesions with very low-background metabolic activity (Figs. 1, 2); of note, patient denied any significant shortness of breath at rest or when walking.

**Fig. 3:**
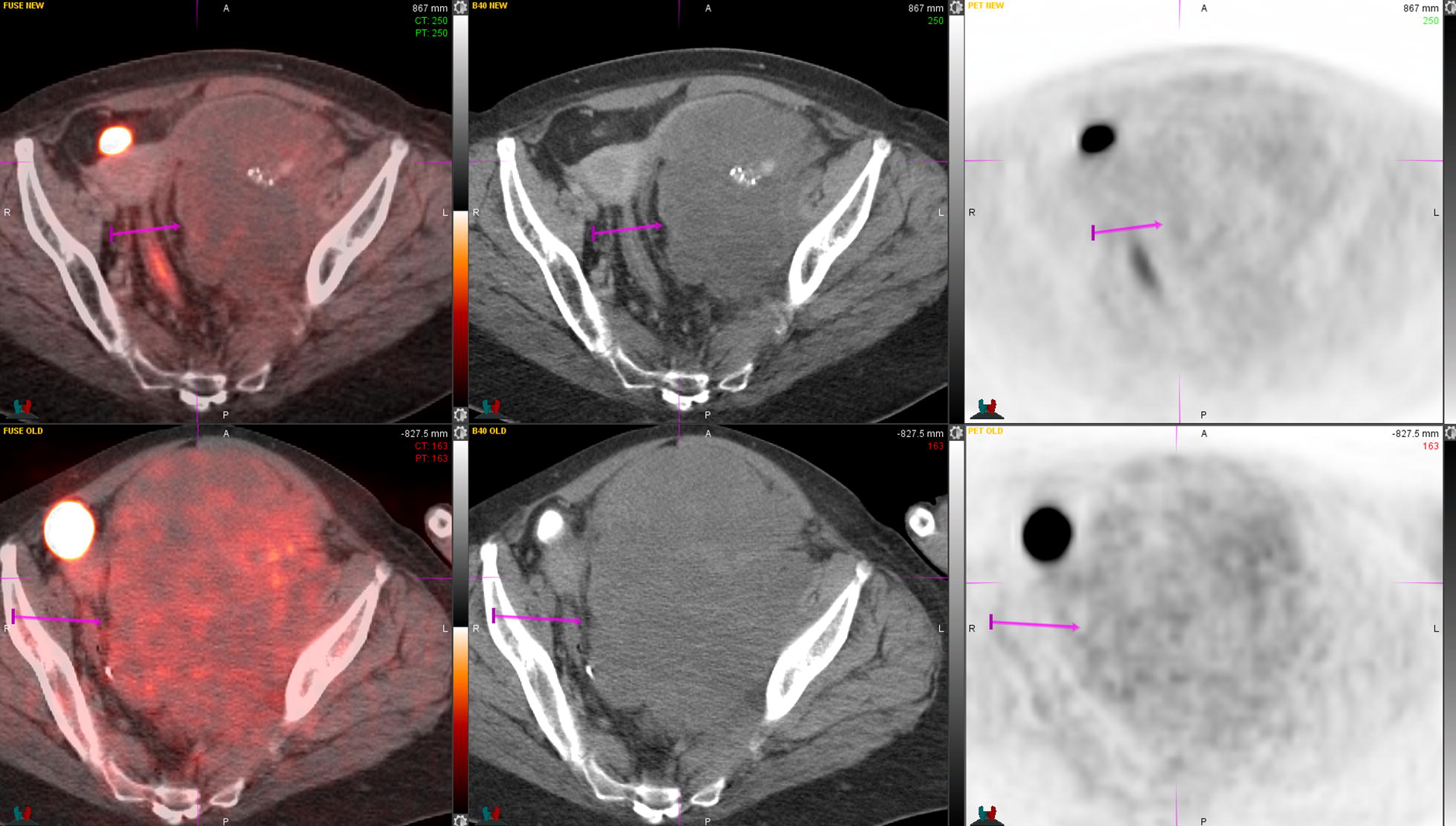
PET/CT before (lower images) and after (upper images) palliative radiation therapy to the pelvis

**Fig. 4:**
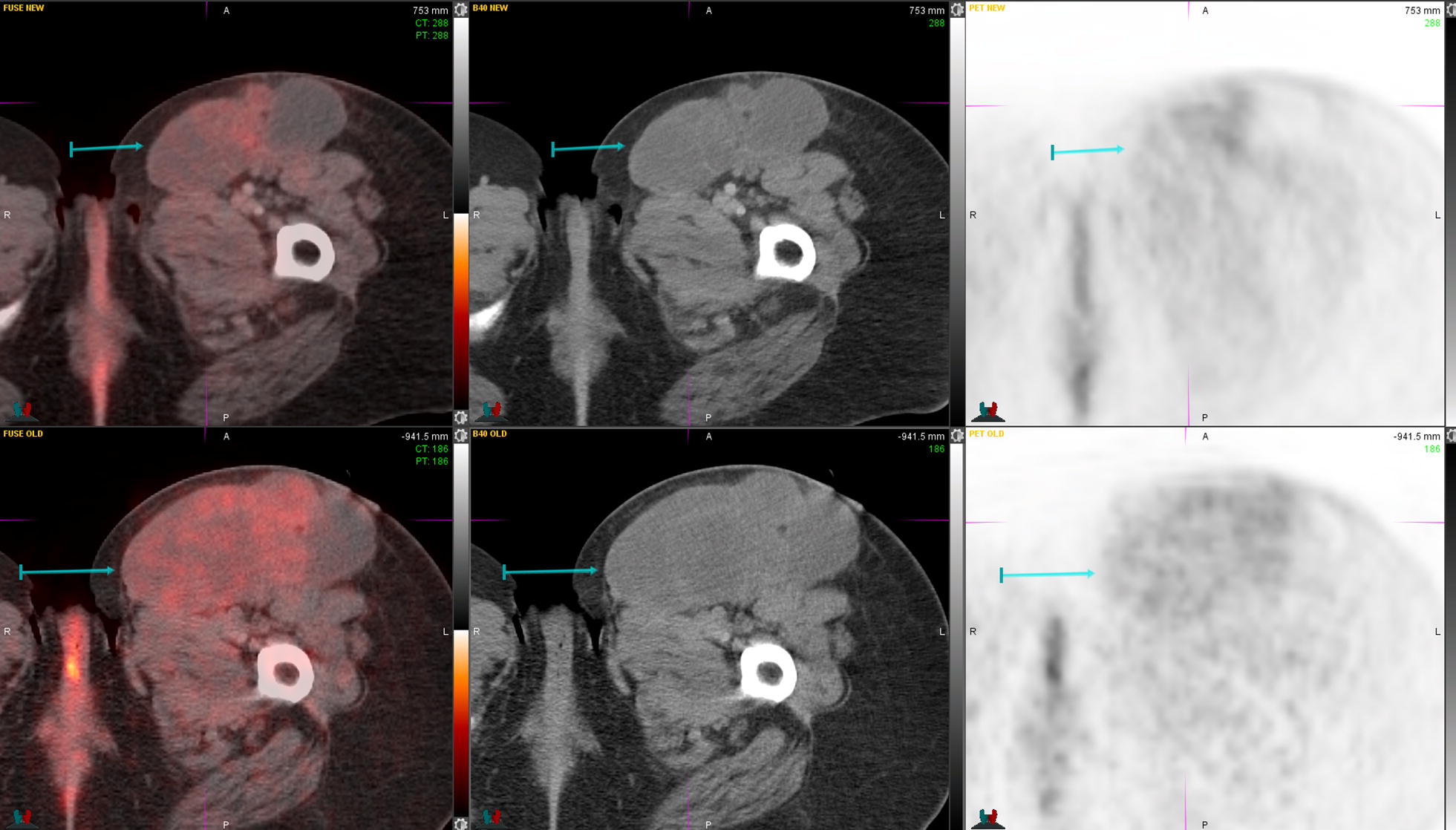
PET/CT before (lower images) and after (upper images) palliative radiation therapy to the left inguinal/thigh area

Seven years after diagnosis, given compression of the left common vein from the tumor, the patient underwent percutaneous transluminal venous angioplasty and stenting with markedly improved blood flow. One year later, given anatomic progression of the pelvic and left inguinal mass, she underwent palliative radiation therapy with photons to a mass in the left pelvis (17.4 × 13.4 cm, SUV = 3.6) and left inguinal area (15.1 × 6.9 cm, SUV = 3.3): 4500 cGy were delivered in 18 fractions. A PET/CT done about 4 months after completing radiation therapy showed a metabolic and anatomic response within the pelvic mass (11.8 × 9.2 cm, SUV = 2.8) and the left inguinal area (12.1 × 4.6 cm, SUV = 2.7; Figs. 3, 4). The patient continued pazopanib 600 mg daily (dose was reduced because of diarrhea) with overall stability of disease during the following 4 years of follow-up. After 3 years, given worsening diarrhea with multiple episodes per day (grade 3 per CTCAE criteria), pazopanib was further dose reduced to 200 mg daily. A new CT scan done after about 5 months since this dose reduction, showed increase in size of multiple large left external iliac, femoral and inguinal nodal masses with diffuse tumor infiltration into the left distal external iliac and femoral veins. Patient underwent angioplasty and stenting of left femoral and iliac veins with good reperfusion.

Patient continued the dose reduced pazopanib that was increased to 400 mg after this procedure; she is not able to tolerate higher doses due to worsening diarrhea. She has an ECOG PS of 1 due to a sense of heaviness to her left lower extremity from chronic edema but no other significant symptoms such as shortness of breath or pain.

## Discussion and conclusions

Extraskeletal myxoid chondrosarcoma is a rare translocation-associated soft tissue sarcoma of uncertain differentiation. More than 90% of EMC have a characteristic translocation that involves the NR4A3 gene on chromosome 9; the most common fusion partner is the EWSR1 gene on chromosome 22, but alternative translocations involving the TAF15, TFG or TCF-12 genes have also been described [1]. The EWSR1–NR4A3 and TAF15 fusion proteins have been characterized as transcription factors but the molecular consequences of the observed translocations have only been partly elucidated. EMC is generally characterized by a quite indolent growth rate but strong tendency to local recurrence and metastatic spread, usually in the lungs. Well-differentiated versus higher grade variants and even dedifferentiated cases have been described [1]. Wide local excision is the only treatment that has shown to be potentially curative. Cytotoxic chemotherapy with drugs commonly used in other sarcomas such as ifosfamide, doxorubicin, cyclophosphamide, docetaxel or gemcitabine has generally shown little or no activity in metastatic EMC. Occasional responses to chemotherapy have been described however: 4 out of 10 patients for example, experienced a partial response to an anthracycline alone or combined to ifosfamide, in a recent series (2/4 PR in patients with metastatic disease) [2–4]. No drug is specifically approved by the FDA for this disease. Check-point inhibitors have occasionally shown clinical activity in other chondrosarcoma subtypes: for example, one PR to first line nivolumab was observed in a patient with a metastatic dedifferentiated chondrosarcoma in a retrospective series and one PR in a phase 2 study with pembrolizumab after prior systemic treatment [5, 6]. In a retrospective report from Kostine et al., PD-L1 expression was associated with the number of tumor-infiltrating lymphocytes and HLA class 1 expression in 11 out of 21 dedifferentiated chondrosarcoma (52%) [7]. Data on PD-L1 expression or other factors that may be potentially predictive of response to checkpoint inhibitors such as tumor mutational burden or microsatellite instability status in EMC is very limited. Davis et al. used a next-generation sequencing approach to genomically profile 6 patients with EMC; similar to other translocation-associated sarcomas and to the case presented here, the mutational profile was limited beyond the pathognomonic translocation. None of the other genomic aberrations found were recurrent or considered clinically relevant. Significant overexpression of RET compared to other types of sarcomas, was observed (p < 0.0002); additionally, the folate receptor was found to be overexpressed in 2 patients [8].

Tyrosine kinase inhibitors may have better activity than chemotherapy in this challenging disease: clinical benefit from sunitinib has been observed in 8 out of 10 patients with the EWSR1–NR4A3 translocation (6 PRs); elevated expression and activation of RET, a known target of sunitinib, was noted in this study [9]. Some studies suggest a more aggressive phenotype and possibly clinical behavior for EMC with alternative fusions but data is limited [9, 10]. A phase 2 trial of pazopanib in NR4A3 positive advanced EMC recently enrolled 24 patients; 1 patient out of 20 had a PR, 17 patients had stable disease and 2 patients had progression. At a median follow-up time of 13 months, the median progression free-survival was 13 months (range 1.6–25.1) with 29% of patients free of progression at 18 months [11]. In line with the data above, our patient had an EWSR1–NR4A3 translocation and experienced prolonged disease stabilization from pazopanib; the presence of additional mutations involving different cancer-related pathways in this case is interesting but no definite conclusions can be drawn. Potential benefits on cancer specific survival (CSS) from external beam radiotherapy (EBRT) has been suggested by a population-based analysis of the SEER database where 172 cases with localized disease were identified; a CSS of 94% versus 85% at 5 years was observed for patients receiving EBRT versus no EBRT at a median follow-up of 33 months (p = − 0.01); also, a trend toward an overall survival benefit associated with EBRT was noted (p = 0.06) [12].

The case described is interesting for the following reasons. First, the patient developed an impressive disease burden in both lungs without significant symptoms such as shortness of breath or cough for several years. Second, this patient experienced prolonged disease control from local treatment with EBRT and systemic treatment with pazopanib, in line with other small series. Third, this patient may have experienced a disease flare in the pelvic area after dose reduction of the TKI, a phenomenon described in in vivo animal models and in patients with renal cell carcinoma [13]. While checkpoint inhibitors have shown a possible therapeutic role in some patients with other types of chondrosarcoma, their use for this particular patient may not be advisable given the low TMB, a factor predictive of response to checkpoint inhibition in other malignancies; also, such immunotherapies could be problematic in this case given the extensive lung involvement and the possibility of pneumonitis, an usual but possible adverse effect. New treatment approaches are needed in this rare sarcoma; a combination of TKIs and checkpoint inhibitors may be worth it to be explored in some types of chondrosarcoma and potentially this subtype. Interestingly, the combination of the TKI sunitinib and nivolumab showed one PR in a patient with chondrosarcoma out of 14 total patients with different histologies in a study recently presented at the ASCO 2018 annual meeting [14].

Enrollments of patients into basket trials may facilitate the development of novel treatment strategies and help selecting patients in a more systematic and hopefully, successful way.

## Abbreviations

EMC: extraskeletal myxoid chondrosarcoma
TKI: tyrosine kinase inhibitor
STS: soft tissue sarcoma
NGS: next generation sequencing
TMB: tumor mutational burden
Mb: megabase
VEGF2: vascular endothelial receptor 2
IGF-R1/IR: insulin-like growth factor 1 receptor/insulin receptor
TKI: tyrosine kinase inhibitor
VEGF: vascular endothelial growth factor
CTCAE: Common Terminology Criteria for Adverse Events
PET/CT: positrone emission tomography/computed tomography
PS: performance status
FDA: Food and Drug Administration
PR: partial response
CSS: cancer specific survival
EBRT: external bean radiotherapy
SEER: surveillance, epidemiology and end results
ASCO: American Society of Clinical Oncology
